# Cerebrospinal Fluid Erythrocyte Burden Amplifies the Impact of PTAU on Entorhinal Degeneration in Alzheimer’s Disease

**DOI:** 10.3390/biom15091300

**Published:** 2025-09-10

**Authors:** Rafail C. Christodoulou, Georgios Vamvouras, Vasileia Petrou, Platon S. Papageorgiou, Rafael Pitsillos, Ludwing Rivera, Evros Vassiliou, Sokratis G. Papageorgiou, Elena E. Solomou, for the Alzheimer’s Disease Neuroimaging Initiative

**Affiliations:** 1 Department of Radiology, Stanford University School of Medicine, Stanford, CA 94305, USA; 2 Department of Mechanical Engineering, National Technical University of Athens, 15772 Zografou, Greece; gvamvouras@mail.ntua.gr; 3 Department of Medicine, University of Ioannina, 45110 Ioannina, Greece; md07010@uoi.gr; 4 2nd Department of Orthopaedic Surgery and Traumatology, Aghia Sophia Pediatric General Hospital, Thivon 3 Street, 15772 Athens, Greece; pplaton24@gmail.com; 5 Neurophysiology Department, The Cyprus Institute of Neurology and Genetics, Nicosia 2371, Cyprus; rafaelp@cing.ac.cy; 6 Department of Medicine, American University of Antigua College of Medicine, St. Johns 1451, Antigua and Barbuda; Ludwingr@auamed.net; 7 Department of Biological Sciences, Kean University, Union, NJ 07083, USA; evassili@kean.edu; 8 1st Department of Neurology, Medical School, National and Kapodistrian University of Athens, Eginition Hospital, 15772 Athens, Greece; sokpapa@med.uoa.gr; 9 Internal Medicine-Hematology, University of Patras Medical School, 26500 Rion, Greece

**Keywords:** Alzheimer’s disease, cerebrospinal fluid, red blood cells, PTAU, entorhinal cortex

## Abstract

Background: Alzheimer’s disease (AD) involves ongoing neurodegeneration, with phosphorylated tau (PTAU) intracellular accumulation closely associated with cortical shrinking. However, not everyone with high PTAU levels shows the same degree of neurodegeneration, implying that other biological stress factors might influence tau’s harmful effects. This research explores whether cerebrospinal fluid erythrocyte burden (CTRED), a marker indicating vascular–CSF barrier disruption and heme toxicity, affects the link between PTAU181 levels and entorhinal cortex atrophy in AD. Methods: We examined 25 observations from 18 patients with AD using a linear mixed effects model. The dependent variable was entorhinal cortex volume, with fixed effects for PTAU, CTRED, and their interaction. Random intercepts accounted for variability within subjects. A cognitively normal (CN) control group was included for comparison. Results: CTRED is significantly associated with reduced entorhinal volume (*p* = 0.005). A notable interaction between CTRED and PTAU was also found (*p* = 0.004), suggesting that higher CTRED enhances PTAU’s atrophic effects. PTAU alone was not a significant predictor. No significant effects were observed in the CN group, which supports the specificity of the disease. Conclusions: CTRED alters the neurotoxic impact of PTAU on the entorhinal cortex in AD, supporting a multi-hit model of degeneration that involves tau pathology and erythrocyte-derived stress. These findings emphasize the clinical importance of vascular–CSF biomarkers in predicting neurodegeneration and guiding targeted treatments.

## 1. Introduction

AD is a neurodegenerative disorder that is associated with advanced progressive dementia [1] and is recognized as its most common cause [2]. Both the prevalence and incidence of AD increase with age [3]. The disease is characterized by gradual neuronal degeneration that leads to progressive memory loss and decline in cognitive functions [2]. Its pathophysiology involves the deposition of extracellular β-amyloid plaques and accumulating PTAU within neurons in neurofibrillary tangles (NFTs) [1].

Regarding tau pathology, two main mechanisms have been proposed to explain its hyperphosphorylation in AD patients. The first states that tau protein undergoes structural modifications that increase its susceptibility to phosphorylation. The second one describes an imbalance in the activity of kinases and phosphatases 1 and 2A, disrupting their normal phosphorylation state. This increased phosphorylation impairs the normal function of tau and leads to microtubule disassociation, resulting in synaptic dysfunction, neuronal death, and consequently leads to dementia [4].

Tau protein is a biomarker in various neurodegenerative diseases, including AD. T-tau and PTAU are the most widely used forms. T-tau reflects generalized neuronal injury and is elevated in AD and other neurodegenerative disorders. In contrast, PTAU is more specifically linked to Alzheimer’s pathology. It has been shown to correlate with cognitive decline even in the preclinical stages of the disease, underscoring its prognostic value in predicting disease progression [5]. T-tau demonstrates high sensitivity for detecting neuronal injury but shows low specificity, limiting its diagnostic utility. A tau-only model shows considerable variability and appears to be insufficient for the reliable differentiation of AD from other neurological conditions [6].

The initial site of tau pathology in AD is the entorhinal cortex (EC), as NFTs first appear in this region [7]. MRI studies have demonstrated volume loss in the EC in cases of mild cognitive impairment, suggesting its value as an early marker of preclinical alterations in AD [8]. Furthermore, the EC is a functional hub connecting the neocortex with the hippocampus by transmitting information via the perforant and temporoammonic pathways. Consequently, disruptions in the EC can impair communication with the hippocampus and lead to broader functional deficits [9].

Another emerging biomarker in AD research is the CTRED. In a previous study we demonstrated that increased erythrocyte presence in the cerebrospinal fluid (CSF) was significantly associated with hippocampal atrophy in patients with Alzheimer’s disease [10]. Specifically, this finding suggested that RBC infiltration into the CSF, likely resulting from microvascular damage, may contribute to neurodegenerative processes by releasing hemoglobin breakdown products, inducing oxidative stress, and inflammation.

Despite growing evidence linking cerebrovascular dysfunction with neurodegeneration in AD, there is a notable lack of studies investigating the interactive effects between PTAU pathology and vascular-related cerebrospinal fluid (CSF) burden, particularly erythrocyte load quantified by CTRED. Given that the entorhinal cortex is one of the earliest regions to exhibit atrophy in AD, it represents a critical focus for understanding the mechanisms driving disease progression. In this study, we hypothesize that CTRED amplifies the detrimental effect of PTAU on entorhinal cortex volume loss, suggesting a synergistic interplay between vascular injury and tau-mediated neurodegeneration in AD. Hence, we have designed a linear mixed effects model (LMM) to correlate the average volume of the entorhinal cortex in AD patients with tau and CTRED values to identify a relationship between the two metrics and volume loss.

## 2. Materials and Methods

### 2.1. Data Acquisition

This study utilized tabular patient metadata and imaging data from the Alzheimer’s Disease Neuroimaging Initiative (ADNI) database (http://adni.loni.usc.edu accessed on 3 June 2025). The ADNI project was launched in 2003 as a public–private partnership with the primary goal of testing whether clinical, imaging, genetic, and biochemical biomarkers can be combined to measure the progression of Mild Cognitive Impairment (MCI) and early AD. All participants gave written informed consent at the time of enrollment for data collection and sharing. The study protocols and consent forms were approved by the institutional review boards (IRBs) of each participating institution.

Only subjects classified as cognitively normal CN or as Alzheimer’s disease patients AD were included in the analysis. Group participants were similar in terms of metabolic and genetic factors (diabetes, hypertension, Age) that could affect CTRED levels to minimize potential confounding. Participants with MCI were excluded to reduce heterogeneity, as ADNI subdivides MCI into early, standard, and late MCI. These transitional states may involve mixed or non-AD pathologies, complicating interpretation. The AD group in ADNI includes only mild cases (MMSE 20–26, CDR 0.5–1), ensuring a more uniform cohort for comparison with controls. Additionally, to avoid bias from extreme values potentially related to procedural difficulties or patient non-cooperation, outliers were systematically excluded from the CTRED dataset prior to analysis. After restricting visits containing T1-weighted MRI scans and relevant clinical measurements, 2885 were deemed useful for analysis.

Subject selection proceeded in two stages. Initially, only individuals with at least two time points that included PTAU181 and CTRED biomarker measurements and an MRI acquired within a reasonable temporal proximity were retained. This ensured that longitudinal trends could be assessed. Participants with only one such visit were omitted. Secondly, MRI scans were filtered based on compatibility with the FreeSurfer software (version 7.4.1). Sequences such as Localizer, Gradwarp, field mapping, scaled images, and N3 scans were discarded due to technical incompatibility with FreeSurfer or incomplete brain coverage. For the patients in the resulting cohort, a comparison was made between the CN and AD groups regarding APOE ε4 carrier status and allele count, clinical severity assessed by the CDR–Sum of Boxes, neuropsychiatric status evaluated by the NPI-Q total score, and comorbidities (hypertension and diabetes) derived from the ADNI Medical History dataset. Following this match statistician match, we ensured that the phenotype of our patients is pretty similar in all the factors that potentially affect CTRED levels, such as age, hypertension, or diabetes. As expected, APOE4 status was much more common in the AD group, as a well-established genetic factor in AD. Since it is also known to influence the integrity of the blood–brain barrier (ΒΒΒ) [11], we acknowledge that this could be a potential biological modifier of CTRED toxicity rather than a confounder. This aligns with our hypothesis that multiple factors converge on tau-related neurodegeneration. Baseline characteristics are summarized in Table 1.

**Table 1:** Baseline demographic, genetic, and comorbidity characteristics of AD vs CN subjects.

Variable	AD (n = 18)	CN (n = 49)
APOE4 carriers (≥1 ε4)	83.3%	26.5%
APOE4 copies		
0 copies	16.7%	73.5%
1 copy	55.6%	24.5%
2 copies	27.7%	2.0%
CDR-SB (mean, SD)	4.31, 2.00	0.02, 0.10
NPI-Q total (mean, SD)	2.56, 3.05	0.35, 0.86
Hypertension	83.3%	77.6%
Diabetes	33.3%	34.7%

Diagnostic labels within the ADNI cohort were assigned based on an integrated medical history evaluation, imaging data (MRI and PET), neuropsychological test MMSE, and molecular biomarkers. Numerous variables were initially considered, but only a subset, particularly PTAU181 and CTRED, demonstrated clinical relevance and statistical significance with entorhinal atrophy. Group demographics are presented in Table 2.

**Table 2:** Demographic distribution of age and sex in CN vs AD groups.

Mean ± Stddev	Cognitively Normal (CN) (49 Subjects)	Alzheimer’s Disease (AD) (18 Subjects)
Years of Age	76.2 ± 4.9	73.2 ± 7.2
Females	25 (51%)	10 (56%)
Males	24 (49%)	8 (44%)

### 2.2. Image Processing and Segmentation

Raw DICOM files were downloaded for each subject. Those were processed to extract left and right entorhinal volumes using the FreeSurfer software [12] (v7.4.1; https://surfer.nmr.mgh.harvard.edu/, accessed on 18 June 2025), which automatically segments cortical and subcortical structures and outputs volumetric metrics, including whole brain volume used for normalization. Each segmentation took 6–8 h; the pipeline was deployed on a Google Cloud Platform Linux-based virtual machine (VM) using an N2-standard configuration (Intel^®^ Xeon^®^ Cascade Lake, 8 vCPUs, 32 GB RAM). This setup allowed parallel processing of 8 scans, maximizing throughput.

The entire workflow was automated via bash scripts and included the following steps:

• Unzip DICOM files and convert to NIFTI
• Loop over all unprocessed scans, processing 8 in parallel:
• Monitor processing outcome (success, failure, or unusable)
• Log result and replace with the following scan in the queue

Scans labeled as “GradWarp,” “Scaled,” “Localizer,” “Field Mapping,” or “N3” were systematically excluded. Moreover, a temporal matching tolerance of up to three months was permitted due to non-identical MRI and clinical visit dates.

Outlier detection followed, and entorhinal volume values below the 5th percentile or above the 95th percentile were removed if deemed biologically implausible. Each subject’s average entorhinal volume (left and right) was normalized using the formula total brain volume (brain vol). EC was selected due to its vulnerability in the early stages of AD, given that our cohort corresponds to mild/early AD. Normalization serves as an internal control to ensure findings are not driven by global brain atrophy but are specific to the entorhinal cortex.

$$norm\_avg\_entorhinal=\frac{rh\_entorhinal+lh\_entorhinal}{2\cdot brainvol}$$

### 2.3. Model Development

A mixed linear model was fitted with random intercepts per subject (subid), using the average entorhinal volume normalized by total brain volume (*norm_avg_entorhinal*) as the dependent variable, and PTAU, CTRED, and their interaction as fixed effects. The model formula can be summarized as:

*norm_avg_entorhinal* ~ *PTAU* * *CTRED*

Or in a more mathematically rigorous way:

$$norm\_avg\_entorhinal=\beta_{0}+\beta_{1}\cdot PTAU_{i}+\beta_{2}\cdot CTRED_{i}+\beta_{3}\cdot \left(PTAU_{i}\times CTRED_{i}\right)+u_{j[i]}+\varepsilon_{\iota }\;$$

where

• i: observation index (row in data)
• $\beta_{0}$: is the intercept (baseline mean when all predictors are zero)
• $\beta_{1},\beta_{2},\;\beta_{3}$: are fixed effect coefficients corresponding to main effects and interaction terms
• $u_{j\left[i\right]}$: is the random intercept for subject j corresponding to observation *i* and accounts for individual-level variability
• $\varepsilon_{i}$: is the residual error term.

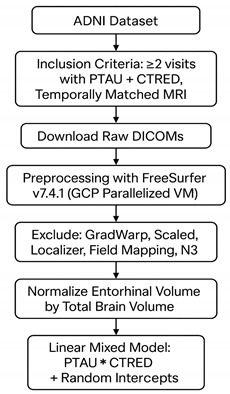


## 3. Results

### 3.1. Model Description

A mixed linear model was fitted with random intercepts per subject (subid), using the average entorhinal volume normalized by total brain volume (*norm_avg_entorhinal*) as the dependent variable, and PTAU181, CTRED, and their interaction as fixed effects. *norm_avg_entorhinal* is defined as:

$$norm\_avg\_entorhinal=\frac{rh\_entorhinal+lh\_entorhinal}{2\cdot brainvol}$$

The model formula can be summarized as:

*norm_avg_entorhinal* ~ *PTAU* * *CTRED*

Or in a more mathematically rigorous way:

$$norm\_avg\_entorhinal=\beta_{0}+\beta_{1}\cdot PTAU_{i}+\beta_{2}\cdot CTRED_{i}+\beta_{3}\cdot \left(PTAU_{i}\times CTRED_{i}\right)+u_{j[i]}+\varepsilon_{\iota }$$

where

• i: observation index (row in data)
• $\beta_{0}$: is the intercept (baseline mean when all predictors are zero)
• $\beta_{1},\beta_{2},\;\beta_{3}$: are fixed effect coefficients corresponding to main effects and interaction terms
• $u_{j\left[i\right]}$: is the random intercept for subject j corresponding to observation *i* and accounts for individual-level variability
• $\varepsilon_{i}$: is the residual error term

### 3.2. Model Results

#### 3.2.1. Interpretation

The analysis (Table 3) showed a statistically significant adverse main effect of CTRED on entorhinal volume, and a statistically significant positive interaction between PTAU181 and CTRED. While PTAU181 alone was not significantly associated with normalized entorhinal thickness, its interaction with CTRED was substantial, indicating an amplification effect. This suggests that the relationship between PTAU and entorhinal atrophy strongly depends on the level of CTRED. It should be noted that in interaction models, main effects reflect conditional relationships (e.g., when CTRED equals a particular value), and their standalone non-significance does not undermine the validity of a significant interaction term that they partake in.

**Table 3:** Mixed linear model results assessing the effects of PTAU, CTRED, and their interaction on entorhinal volume in the AD group.

Predictor	Coefficient	Std. Error	*z*-Value	*p*-Value	95% CI Lower	95% CI Upper
Intercept	0.002	<0.001	+9.023	<0.001	+0.001	+0.002
PTAU	<0.001	<0.001	−0.815	0.415	<0.001	<0.001
CTRED	<0.001	<0.001	−2.829	0.005	<0.001	<0.001
PTAU: CTRED	<0.001	<0.001	+2.845	0.004	<0.001	<0.001

#### 3.2.2. Interpretation for CN Group

In the CN control group, the mixed linear model revealed no statistically significant associations between normalized average entorhinal volume and the predictors PTAU, CTRED, or their interaction. All *p*-values were well above conventional significance levels, indicating that the relationship observed in the AD group does not appear in the CN cohort. This supports the specificity of the PTAU-CTRED interaction effect on entorhinal volume reductions in subjects diagnosed with Alzheimer’s Disease.

### 3.3. Model Prediction Plots and Interpretation

**Figure 1:**
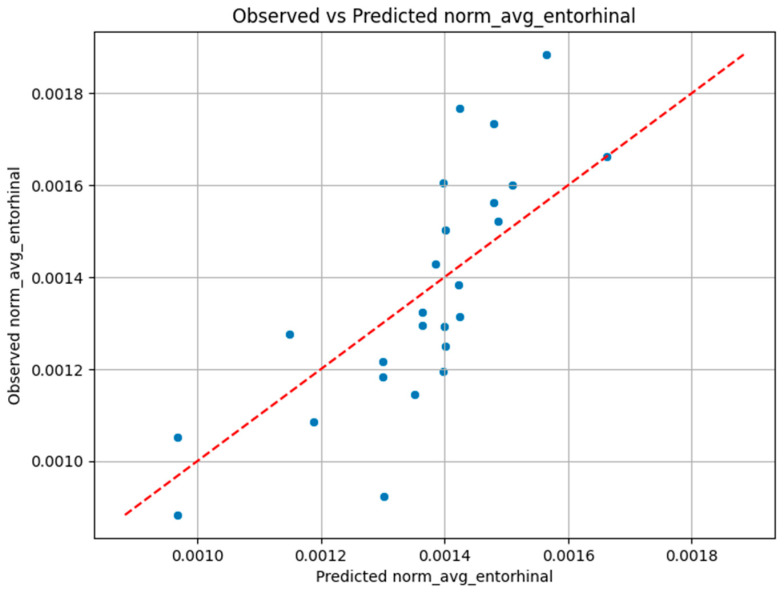
Observed vs. Predicted entorhinal volume for the AD group.

The plot in Figure 1 shows the AD group’s predicted versus observed entorhinal volumes. The biomarkers show significant correlation with volume, and the model effectively captures this variation, although biological heterogeneity introduces slight variability in the predictions.

**Figure 2:**
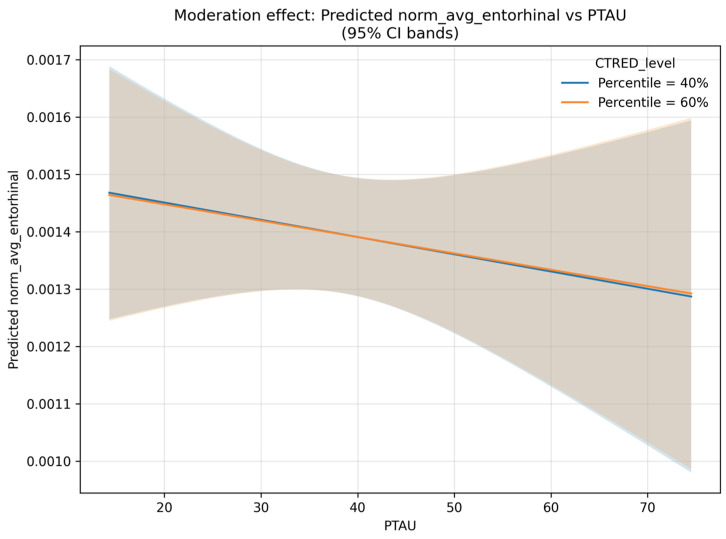
Interaction between PTAU181 and entorhinal volume for different levels of CTRED.

The interaction plot (Figure 2) displays model-predicted normalized entorhinal thickness as a function of PTAU181 at two illustrative CTRED levels (p40 and p60), with shaded 95% confidence bands from the fixed effects. The two lines are close because the selected CTRED percentiles are adjacent, but their slopes differ, meaning that the PTAU slope is slightly less negative at higher CTRED (p60), which is consistent with a significant PTAU × CTRED interaction in the mixed-effects model (z = 2.845, *p* = 0.004). Confidence bands widen toward the extremes of PTAU, reflecting fewer observations in those regions.

## 4. Discussion

### 4.1. Summary of the Main Findings

Our study demonstrates that cerebrospinal fluid red blood cell burden significantly amplifies the relationship between phosphorylated tau and entorhinal cortex atrophy in patients with AD. While PTAU181 alone was not significantly correlated with entorhinal volume, elevated CTRED revealed a statistically significant negative relationship, indicating that PTAU’s neurotoxic effects are amplified in the context of erythrocyte-related CSF toxicity.

In the present study, we analyzed PTAU181, the isoform quantified in the ADNI dataset. PTAU181 has been extensively validated as a biomarker for AD, with strong associations with both neurodegeneration and cognitive decline. Mechanistically, phosphorylation at threonine 181 destabilizes tau’s microtubule-binding function and promotes neurofibrillary tangle formation, linking it directly to AD pathophysiology [13,14]. Longitudinal analyses have shown that plasma PTAU181 can predict AD neuropathology years before clinical diagnosis, supporting its role as an accessible biomarker for early disease detection [14].

However, it is essential to note that PTAU217 and PTAU231 have recently emerged as potential biomarkers. Comparisons show that PTAU217 outperforms PTAU181 (AUC 0.99 vs. 0.96) in diagnosing AD, and meta-analyses have found that pTau217 is more accurate in predicting conversion from MCI to AD [15,16].

This is the first study to model the interaction between PTAU and erythrocyte toxicity in CSF using neuroimaging and fluid biomarkers, supporting a context-dependent framework for tau-mediated atrophy. These findings support a multi-hit model of neurodegeneration in AD, where tau pathology alone may be insufficient to induce significant structural damage unless compounded by additional insults such as heme toxicity and microglial activation.

### 4.2. Tau Pathology and Entorhinal Cortex Vulnerability

PTAU plays a central pathogenic role in AD, primarily as the core component of intraneuronal neurofibrillary tangles (NFTs), which disrupt neuronal function and structure [16]. Under normal physiological conditions, tau stabilizes microtubules in neurons; however, in AD, tau becomes abnormally hyperphosphorylated, dissociates from microtubules, and aggregates into paired helical filaments (PHFs) that form NFTs [16]. These aggregates impair axonal transport and synaptic integrity, leading to progressive neuronal dysfunction and neurodegeneration [13]. PTAU is also considered a hallmark biomarker of AD, strongly associated with both neurodegeneration and cognitive decline. It reflects tau hyperphosphorylation, aggregation, and its subsequent prion-like spread across connected brain regions, especially within the medial temporal lobe [16,17]. Its neurotoxic effects are amplified in the presence of synergistic stressors such as amyloid deposition, inflammation, vascular dysfunction, and oxidative damage, supporting a multi-hit model of AD pathogenesis. Among these, EC is one of the earliest and most vulnerable targets of tau pathology, even at the preclinical stages of AD. Functional neuronal studies show that EC neurons exhibit early hyperexcitability due to parvalbumin-interneuron dysfunction, which may initiate or amplify tau deposition. [18] Besides functional vulnerability, the EC is prone to iron-mediated oxidative damage. Due to low antioxidant capacity, the EC and hippocampal CA1 are among AD’s most ROS-sensitive brain regions [19]. Iron generates free radicals through the Fenton chemistry reaction, and the produced oxidative stress destabilizes iron regulation, creating a feed-forward loop of toxicity [20]. Thus, oxidative stress, iron overload, and inflammation amplify neurotoxicity in AD.

Although PTAU was negatively associated with entorhinal cortex volume in our sample, this relationship did not reach statistical significance, likely due to limited statistical power given our small sample size. Nevertheless, this aligns with the hypothesis that tau’s neurotoxic effects may not manifest uniformly across individuals but instead require the presence of additional pathological stressors [21,22]. In particular, prior work has shown that vascular dysfunction, such as impaired cerebral perfusion or blood–brain barrier disruption, can synergistically amplify tau-related pathology, accelerating atrophy and cognitive decline [21].

In our study, PTAU 181 showed only a weak, non-significant association (*p* = 0.415) with EC volume, but its interaction with CTRED produced a significant amplification effect (*p* = 0.004). This supports a context-dependent model of tau-mediated neurodegeneration, where PTAU alone may be insufficient to drive structural atrophy unless compounded by secondary insults such as oxidative stress and inflammation.

The EC plays a critical role in memory formation, spatial navigation, and olfactory processing, all commonly disrupted in AD. Functionally, it is a hub between the hippocampus and neocortex, encoding and consolidating new memories [23]. The medial EC is responsible for spatial processing, while the lateral EC is responsible for object recognition and sensory integration [24]. Therefore, its central role in the memory circuit facilitates an easier understanding of why even subtle atrophy in the EC can result in significant cognitive deficits.

### 4.3. Microvascular Dysfunction and Cerebrospinal Fluid (CSF) Erythrocyte Toxicity

**Figure 3:**
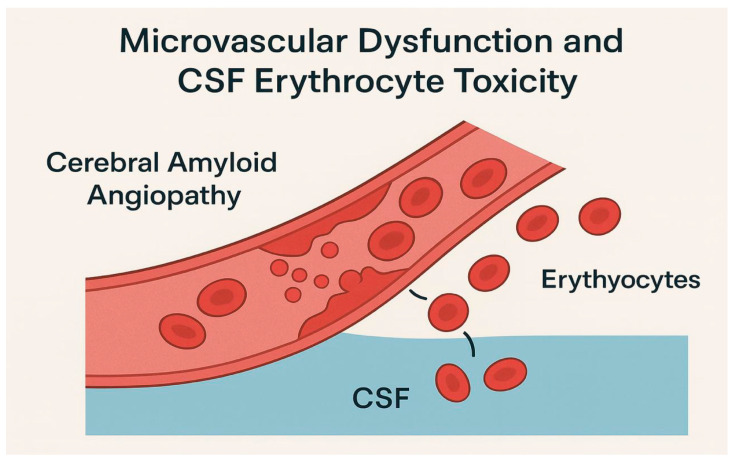
Disruption of the vessels allowing erythrocytes to flow into the CSF and cause toxicity via the mechanisms described in the text.

In AD, cerebral amyloid angiopathy frequently accompanies amyloid pathology, which connects the deposition of β-amyloid in the walls of small to medium-sized cerebral blood vessels [25]. This process weakens the integrity of the vessel, contributing to microhemorrhages and BBB, allowing RBCs to enter the CSF, as illustrated in Figure 3. Therefore, differentiating amyloid-positive from amyloid-negative individuals is crucial. A recent meta-analysis showed that blood-based PTAU181 can predict the amyloid status of AD patients [14]. Predicting amyloid burden can help enroll AD patients into therapeutic trials that can stabilize the BBB or iron chelation therapies to reduce the toxicity of amyloid-related angiopathy micro hemorrhages. Notably, tau-negative amyloid-positive patients have higher permeability than the amyloid-positive tau-positive ones, but vascular factors drive the permeability when stratified according to Apoe status [11]. Tau and vascular factors both support the findings of CTRED toxicity, as hypertension can cause not only BBB impairment but also microhemorrhages that affect neurons. An overview of PTAU and CTRED roles, their regional impact, and interactive effects on neurodegeneration is summarized in Table 4.

**Table 4:** Summary of Imaging Biomarkers and their effects on the progression of degeneration, regarding the region that is mostly affected and the interactions between the molecules.

Biomarker	Pathological Role	Region Affected	Interaction Effect
Phosphorylated Tau (PTAU)	Promotes tau aggregation and neurofibrillary tangle formation, impairing axonal transport and causing neurodegeneration.	Primarily medial temporal lobe structures, especially the entorhinal cortex and hippocampus.	Alone, not always significantly associated with volume loss.
CSF Erythrocyte Burden (CTRED)	Indicates vascular–CSF barrier disruption releases heme iron, causing oxidative stress and inflammation.	Regions with early vascular compromise modulate tau’s effect on the entorhinal cortex.	Amplifies PTAU-related atrophy when elevated, supporting a multi-hit model of neurodegeneration.

### 4.4. Microglial Response to Hemolysis

**Figure 4:**
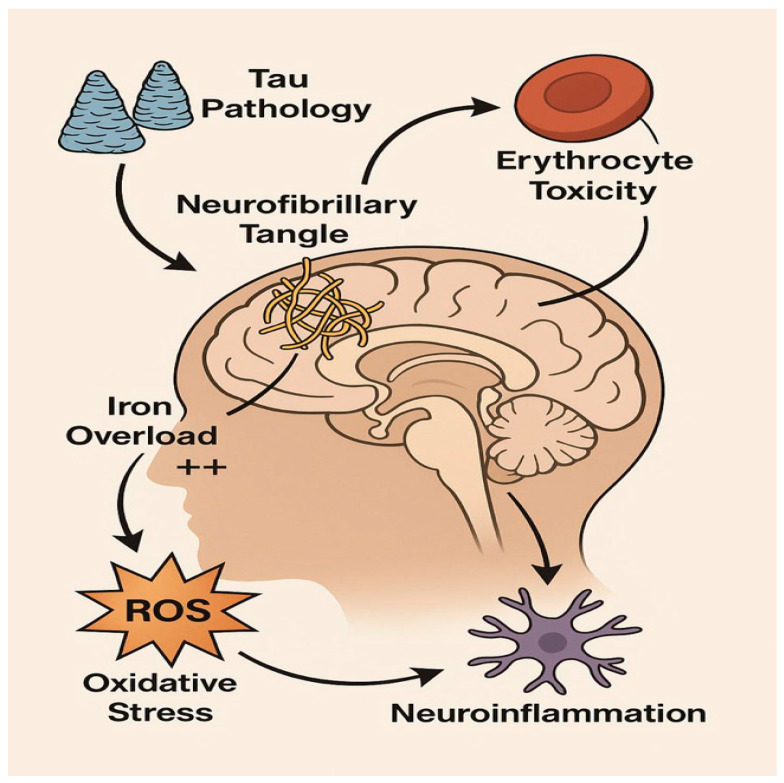
After the breakdown of erythrocytes, heme is released into the CSF, and hence iron homeostasis is disrupted, leading to the formation of reactive oxygen species (ROS), facilitating a sequence of events that cause further hyperphosphorylation of tau and sustain the neuroinflammation that is already present in Alzheimer’s disease.

Extravasated erythrocytes release heme as part of their metabolic process, a danger-associated molecular pattern (DAMP) that can bind to innate immunity receptors and trigger inflammation and oxidative stress [26]. Recent work by Schallner et al. demonstrated that heme oxygenase-1 (HO-1) microglial expression is critical for erythrophagocytosis and detoxification, protecting neurons from injury. In humans with subarachnoid hemorrhage (SAH), increased expression of HO-1 correlates with hematoma volume and inflammatory cytokines [27]. Moreover, erythrolysis-induced secondary brain injury is an established mechanistic effect in hemorrhagic conditions, highlighting that hemolytic products like heme and iron are highly neurotoxic [28]. These byproducts are typically mitigated by microglial erythrophagocytosis (Figure 4). In the context of SAH, neutrophils release lactoferrin. This iron-binding glycoprotein facilitates the efferocytosis of apoptotic RBC and sequestration of toxic Fe3+ [29]. Microglia cells are the brain’s primary immune responders and can adapt diverse phenotypes after injury. While erythrophagocytosis aids in chronic conditions, blood-derived factors can chronically activate it, leading to sustained neuroinflammation. In traumatic brain injury (TBI) and hemorrhagic diseases of the brain, such activation is linked to neuronal dysfunction and cell death [30]. Pre-clinical findings further support the central role of microglia in detoxifying hemolytic byproducts after cerebral hemorrhage [31]. TRIOL is a neuroprotective steroid that, in a Phase II clinical trial, showed promising results as it was shown to enhance microglia erythrophagocytosis via CD36 and promote hemoglobin scavenging through CD163, while reducing inflammatory output and activating the Nrf2 antioxidant pathway [32]. In AD, where microglia are already impaired, the additional burden of clearing CSF RBC may worsen the dysregulation and the inflammatory situation. For instance, microglial proliferation and activation around the amyloid plaques lead to further plaque development, contributing to amyloid-related angiopathy. In addition, this dysfunction mediates synapse loss, worsens tau spread, and simultaneously releases inflammatory mediators [33]. As a result, this could exacerbate neuroinflammation, oxidative stress, and neurodegeneration. These findings underscore the critical role of rebalancing microglia phenotype toward clearance and repair, particularly in pathological settings like AD, where both vascular and inflammatory stressors are met. This provides a plausible explanation for our findings as elevated CTRED intensifies tau-mediated entorhinal degeneration by overwhelming microglial clearance capacity, reinforcing the multi-hit model of AD neurodegeneration.

### 4.5. Iron Metabolism and Oxidative Damage

Building on these findings, iron dyshomeostasis has become a key factor in neurodegeneration, especially in areas like the EC), which is prone to early pathological changes [18,19]. Usually, intracellular iron balance is tightly controlled by proteins such as divalent metal transporter 1 (DMT1), ferroportin (FPN1), and ferritin [34]. However, in Alzheimer’s disease, this balance is disrupted. Iron builds up inside cells, particularly within astrocytes and microglia, producing excessive reactive oxygen species (ROS) through the Fenton reaction [32]. These ROS cause lipid peroxidation, protein damage, and tau hyperphosphorylation, further driving structural deterioration and cognitive decline [34], as depicted in Figure 4.

### 4.6. Ferroptosis Susceptibility in the Entorhinal Cortex

Recent transcriptomic studies support the hypothesis that the entorhinal cortex (EC) is particularly vulnerable to ferroptosis, an iron-dependent, non-apoptotic cell death. In AD brains, EC astrocytes show altered expression of ferroptosis-related genes like SAT1, FTH1, and GPX4, suggesting they are more prone to iron-driven lipid peroxidation [35]. Reduced GPX4 detoxifies lipid hydroperoxides, lowers antioxidant defenses, and increases ROS buildup [36]. These molecular markers and our imaging results suggest that heme and iron from erythrocytes in the CSF may synergize with existing tau pathology to overwhelm neuronal compensatory mechanisms. This leads to localized atrophy in vulnerable regions such as the EC. Our findings align with this vulnerability of EC and can serve as the base for treatments that restore iron balance, such as iron chelators, boosting HO-1, or ferroptosis inhibitors, might be especially effective in slowing disease progression in early AD patients with high CSF erythrocyte levels and PTAU. The mechanisms by which CTRED contributes to neurodegeneration, including heme toxicity, iron overload, microglial activation, and ferroptosis, are summarized in Table 5.

**Table 5:** Mechanisms of Neurodegeneration Linked to CSF RBC Burden.

Mechanism	Description	Implications in Alzheimer’s Disease
Heme Toxicity	RBC breakdown releases heme into CSF, promoting oxidative stress via the Fenton reaction.	Exacerbates neurodegeneration when tau pathology is present.
Iron Overload	Iron accumulation disrupts redox balance, leading to lipid peroxidation and cell death.	Correlates with PTAU-related cortical thinning in the entorhinal cortex.
Microglial Activation	Triggered by blood products, microglia become overactive, secreting pro-inflammatory cytokines.	May fail to adequately clear hemolytic byproducts, contributing to sustained inflammation and worsening of AD pathology.
Ferroptosis	Iron-dependent, non-apoptotic cell death pathway driven by lipid peroxidation and reduced GPX4 activity.	Particularly affects entorhinal astrocytes, contributing to early cortical atrophy in AD.

### 4.7. Comparison to Previous Literature

Our findings expand on existing knowledge of tau-related neurodegeneration by including the amplification role of cerebrospinal fluid erythrocyte toxicity. While PTAU has been strongly associated with medial temporal lobe atrophy, including in the hippocampus and entorhinal cortex, most previous studies have focused on its primary effects without considering potential modifiers. In contrast, our results indicate that erythrocyte-derived CSF toxicity may significantly enhance tau-induced damage, supporting a multi-hit model of neurodegeneration.

This context-dependent effect aligns with our prior research, which showed a negative link between CTRED and hippocampal volume [10]. We now expand this relationship to the entorhinal cortex and demonstrate that the interaction between CSF RBC and PTAU significantly affects structural atrophy, an aspect not previously explored.

Several studies have shown that elevated PTAU predicts greater regional brain atrophy. For example, La Joie R et al. (2020) found that tau-PET signal is strongly associated with longitudinal volume loss across medial temporal structures [37], while Hanseeuw B. et al. (2021) demonstrated that entorhinal-tau burden closely tracks hippocampal atrophy independently of amyloid status [38]. In addition, a recent meta-analysis confirmed that plasma PTAU181 reliably differentiates amyloid-positive from amyloid-negative individuals across the AD continuum, including dementia, supporting the robustness of the association beyond MCI [39]. However, these investigations did not consider the moderating effects of CSF erythrocyte burden or related neurotoxic influences. Our findings may help explain why PTAU does not consistently predict atrophy and highlight the critical role of CSF-derived stressors like heme and iron in modulating tau’s neurotoxic potential.

### 4.8. Clinical Implications

These findings highlight the importance of monitoring cerebrospinal erythrocyte burden as a potential factor influencing tau-related neurodegeneration. Clinically, tau biomarkers alone might not fully assess atrophic risk in early AD unless combined with oxidative or vascular stress measures. If confirmed in larger groups, CTRED could be used to identify tau-positive individuals at the most significant risk for structural decline.

This work also paves the way for precisely timed interventions: anti-tau therapies or vascular stabilizers might need to be administered earlier in individuals with elevated CTRED. Furthermore, it suggests the potential for developing treatments that target heme clearance, oxidative stress, and iron metabolism to prevent synergistic neuronal damage in tauopathies.

### 4.9. Limitations

The main limitation of our study is its small sample size (n = 18), which could reduce statistical power and hinder the detection of subtle effects. Furthermore, MRI scans were collected using different scanner vendors and protocols, potentially causing variability in image quality and volumetric measurements. This variability impacted the consistency of the structural data used in our analyses. Lastly, CTRED indicates erythrocyte toxicity and BBB dysfunction, but we did not measure heme, iron, oxidative stress, or ferroptosis-related factors.

### 4.10. Future Directions

Future research should focus on validating these interaction effects in larger, independent groups across the AD and MCI spectrum, since it represents an essential intermediate state between our comparison groups. Long-term studies in bigger and independent cohorts are necessary to explore the temporal relationships between PTAU, CTRED, and entorhinal atrophy. Simultaneously, developing direct and quantitative measures of CSF heme, iron, and oxidative stress biomarkers will improve our mechanistic understanding of erythrocyte-induced neurotoxicity. Besides fluid markers, imaging techniques such as quantitative susceptibility mapping (QSM) could serve as non-invasive indicators of iron accumulation. Machine learning models applied to neuroimaging and clinical data also predict CTRED levels without requiring lumbar punctures. Investigating ways to enhance CSF clearance pathways or stimulate microglial erythrophagocytosis could reduce tau-related neurodegeneration and lead to new therapeutic strategies. Our findings indicate that targeting iron metabolism and neuroimmune dysfunction may be essential in altering disease progression in tauopathies.These clinical and translational implications of CTRED and PTAU interactions are summarized in Table 6.

**Table 6:** Clinical and Translational Implications.

Key Finding	Clinical Implication	Translational Application
CTRED is significantly associated with entorhinal cortex atrophy	Monitoring CSF RBC levels may help predict neurodegeneration risk	Use CTRED in biomarker panels for early detection or disease staging
PTAU alone is not predictive of atrophy	PTAU should not be used in isolation for prognosis	Develop composite biomarker models including PTAU and vascular–CSF markers
CTRED and PTAU interact to predict greater neurodegeneration	Dual-marker models may better stratify patients for targeted therapies	Test antioxidant, anti-ferroptosis, or vascular stabilizing therapies in PTAU+/CTRED+ patients
No significant effects observed in CN controls	Supports the specificity of vascular-tau interaction in AD rather than normal aging	Tailor therapeutic interventions to high-risk biomarker profiles.

## 5. Conclusions

This study shows CTRED significantly strengthens the link between PTAU and entorhinal cortex atrophy in AD. While PTAU alone had a weak correlation with entorhinal volume, its interaction with high CTRED revealed a strong negative relationship, highlighting the role of vascular and oxidative factors in influencing tau pathology. Notably, this connection was not seen in CN controls, emphasizing the specific interaction in AD and supporting a multi-hit model of neurodegeneration.

Our results extend previous evidence connecting tau pathology to medial temporal lobe atrophy by identifying CTRED as a new factor that modifies PTAU toxicity. This may be mediated by erythrocyte breakdown products like heme and iron, which cause oxidative stress, hinder microglial clearance, and increase vulnerability to ferroptosis in the entorhinal cortex. These findings could explain inconsistencies in past studies where tau biomarkers alone did not reliably predict structural decline, and they highlight the importance of considering vascular–CSF interactions in AD progression models.

This work suggests that monitoring CTRED alongside tau biomarkers could be valuable. Patients with high CTRED might be at increased risk for rapid cortical atrophy and cognitive decline. Recognizing these individuals may improve patient selection for trials and inform personalized treatment strategies. Interventions targeting iron toxicity, enhancing CSF clearance, or restoring microglial balance may benefit this subgroup.

However, the results have limitations, including a small sample size and indirect measures of erythrocyte toxicity. Future research with larger, earlier-stage cohorts, measuring heme, iron, and oxidative stress directly, and using advanced imaging methods like QSM, will be essential to validate and expand these findings. Long-term studies are especially needed to see if CTRED can predict disease progression and be a useful prognostic biomarker.

In summary, our research provides the first evidence that vascular–CSF toxicity interacts with tau pathology to cause region-specific neurodegeneration in AD. Combining molecular biomarkers with vascular markers brings us closer to understanding disease mechanisms and developing personalized, targeted treatments for AD.

## Data Availability

The data presented in this study are available on request from the Alzheimer’s Disease Neuroimaging Initiative (ADNI) database: http://adni.loni.usc.edu accessed on 3 June 2025. Access to the data requires registration and compliance with ADNI’s data use agreement. The authors did not generate any new datasets in this study.

## Abbreviations

The following abbreviations are used in this manuscript:

AD	Alzheimer’s Disease
AB	Amyloid Beta
APC	Article Processing Charge
CN	Cognitively Normal
CSF	Cerebrospinal Fluid
CTRED	Cerebrospinal Fluid Erythrocyte Burden
DAMP	Danger-Associated Molecular Pattern
DICOM	Digital Imaging and Communications in Medicine
EC	Entorhinal Cortex
GPX4	Glutathione Peroxidase 4
HO-1	Heme Oxygenase-1
LMM	Linear Mixed Effects Model
MCI	Mild Cognitive Impairment
MMSE	Mini-Mental State Examination
MRI	Magnetic Resonance Imaging
NFT	Neurofibrillary Tangle
PET	Positron Emission Tomography
PHF	Paired Helical Filaments
PTAU	Phosphorylated Tau
QSM	Quantitative Susceptibility Mapping
RBC	Red Blood Cell
ROS	Reactive Oxygen Species
SAH	Subarachnoid Hemorrhage
TBI	Traumatic Brain Injury
T-tau	Total Tau
VM	Virtual Machine
XAI	Explainable Artificial Intelligence
